# Enhanced Bruton’s tyrosine kinase activity in the kidney of patients with IgA nephropathy

**DOI:** 10.1007/s11255-020-02733-2

**Published:** 2021-01-03

**Authors:** Jie Wei, Yan Wang, Xiangming Qi, Yonggui Wu

**Affiliations:** Department of Nephrology, The First Affiliated Hospital, Anhui Medical University, No. 218, Jixi Road, Hefei, Anhui 230032 People’s Republic of China

**Keywords:** IgA nephropathy, Bruton’s tyrosine kinase, Macrophage, NF-κb, Oxford classification, Katafuchi semi-quantitative score

## Abstract

**Purpose:**

Bruton’s tyrosine kinase (BTK) is a vital biological molecule that contributes to immune regulation. Previous studies have showed that BTK can be detected in patients with lupus nephritis and rheumatoid arthritis. However, the role of BTK in IgA nephropathy (IgAN) has not yet been elucidated. The purpose of this research was to investigate the role of BTK activation in macrophages in IgAN.

**Methods:**

Peripheral blood and renal tissue samples were collected from 63 patients with IgAN, and peritumoral normal tissues were collected from 20 patients after surgical resection of renal tumor for use as control. Additionally, 20 healthy volunteers were recruited as control. The levels of BTK, CD68, phosphorylated BTK (pBTK), phosphorylated NF-κB (p-NF-κB p65), tumor necrosis factor (TNF)-α, interleukin (IL)-1β, and monocyte chemotactic protein (MCP)-1 were measured by immunohistochemistry (IHC), real-time polymerase chain reaction (RT-PCR), western blotting, and enzyme-linked immunosorbent assay (ELISA).

**Results:**

Compared to peritumoral normal tissues, the expression levels of CD68 and BTK were significantly increased in IgAN group (*p* < 0.001) and the differences between M0 and M1, E0 and E1, S0 and S1, T0 and T1-2, C0 and C1-2 were statistically significant in the updated Oxford Classification (*p* < 0.05). Also, CD68 and BTK were positively correlated with Katafuchi semi-quantitative glomerular and tubulointerstitial scores (*r* = 0.580, 0.637 and 0.442, 0.489, respectively, *p* < 0.05). The expression of BTK was significantly higher in C3b- and C4d-positive renal tissues of patients with IgAN (*p* < 0.05). In addition, BTK was positively correlated with 24-h urine protein, serum creatinine levels (*r* = 0.456 and 0.453, respectively, *p* < 0.001), and negatively correlated with serum albumin (*r* = 0.357, *p* < 0.05). The intensity of expression of pBTK and p-NF-κB p65 was observably increased in renal tissues and monocytes of patients with IgAN compared to the control group. The results of IHC, RT-PCR, and ELISA indicated that the levels of TNF-ɑ, IL-1β, and MCP-1 were markedly increased in the IgAN group (*p* < 0.05).

**Conclusion:**

The results of this study indicate that activation of BTK in macrophages may play an important role in promoting the progression of renal inflammation in IgAN.

## Introduction

Immunoglobulin A nephropathy (IgA nephropathy, IgAN) is the most common form of primary glomerulonephritis worldwide [1]. The region of Asia has a high incidence of IgAN. A study showed that IgAN accounts for 38–58% of the patients with renal biopsy in China and the trend is increasing every year [2]; approximately 20–40% of these patients would progress to chronic renal failure within 20 years [3, 4]. However, so far, the etiology and pathogenesis of IgAN have remained unclear. Previous basic studies and clinical observations have shown that immune inflammatory response is the core link in the pathogenesis of IgAN, which is closely related to the onset, aggravation, and progression of the disease [5, 6]. In IgAN, kidney damage begins in mesangial cells and includes deposition of immune complexes that activate the proliferation of mesangial cells and induce them to produce various cytokines and chemokines, which are released in the extracellular environment. These inflammatory cytokines are transported to the glomerular interstitium through glomerular filtration and blood circulation to activate glomerular epithelial cells. This process then leads to a cascade of amplifying reactions that promote glomerular mesangial and extracapillary hyperplasia, ultimately leading to proteinuria and glomerular fibrosis [7, 8]. The activated mesangial cells trigger two mechanisms that aggravate renal injury in patients with IgAN; one is infiltration of the renal tubulointerstitium by monocytes and macrophages and the other is damage to the tubulointerstitium caused by exposure of the tubular lumen to albumin [9]. Therefore, revealing the pathogenesis of IgAN is particularly important for improving the prognosis of patients with IgAN.

Bruton’s tyrosine kinase (BTK) is a crucial biological molecule in B-cell antigen receptor (BCR)-mediated signal transduction pathway [10], and it is mainly expressed in the cytoplasm of B lymphocytes, mast leukocytes, monocytes, and other bone marrow cells. There are various receptors downstream of BTK, which participate in various signaling pathways and play an important role in cell proliferation, differentiation, cytokine synthesis, antigen presentation, and other processes [11–13]. An increasing number of evidences also suggest that BTK plays a variety of roles in monocytes of the innate immune system, especially in dendritic cells and macrophages [14]. For example, BTK down-regulates TLR-mediated inflammatory response in human primary innate immune cells. Besides, activated BTK blocks the activity of NF-κB. In tumor and cerebral ischemia/reperfusion animal models, Btk is activated in infiltrating macrophages/neutrophils, suggesting that Btk might have immunosuppressive and tumor-promoting effects, and that inhibiting Btk could prevent brain injury [15–18]. In summary, these data suggest that BTK has become an important target for innate immune regulation, and that limiting BTK activity is essential for preventing autoimmune diseases.

Several articles have reported increased BTK expression in B cells of lupus nephritis, rheumatoid arthritis, and other diseases. However, the role of BTK in IgAN has not been explored. Therefore, in this study, we have discussed the potential mechanism of BTK in the progress of IgAN.

## Materials and methods

### Study subjects

From March to August 2019, 63 patients hospitalized for primary IgAN diagnosed based on pathology of renal biopsies examined in the Nephrology department of the First Affiliated Hospital of Anhui Medical University were recruited as IgAN group. The IgAN group comprised 23 men and 40 women. Pathology scores were based on the Updated Oxford Classification (MEST-C score) [19] and Katafuchi semi-quantitative score [20]. No patient had taken hormones or immunosuppressants before the beginning of this study. In addition, patients with secondary nephropathy such as chronic hepatitis, autoimmune diseases, systemic vasculitis, metabolic diseases, tumors, etc. were excluded from the study. Moreover, patients with abnormal liver function, cardiovascular diseases such as cardiac insufficiency, malignant hypertension, or arrhythmia, infection or stress, and pregnant or lactating women were excluded from the study.

Meanwhile, 20 samples of renal tissue (2 cm) adjacent to the lesion after the surgical resection of renal tumor in the Urology department of our hospital were collected as the control group 1. Besides, 20 age- and gender-matched healthy volunteers were recruited as the control group 2 (no previous history of diabetes, hypertension, cardiovascular disease, liver disease, and kidney disease).

This study was approved by the ethics committee of the First Affiliated Hospital of Anhui Medical University (Ethical Code: Pj2018-13-10), and all subjects voluntarily signed the informed consent form.

### Collection of renal tissue specimens

After definite diagnosis of IgAN by immunofluorescence and electron microscopy, wax blocks were sectioned for later use. The peritumoral tissues were dehydrated and embedded in paraffin to make wax blocks. The peritumoral tissues were confirmed as normal kidney tissues by a pathologist. The thickness of the paraffin sections was 3 µm.

### Plasma conservation and isolation of peripheral blood mononuclear cells

About 10 mL of fasting peripheral blood sample was collected from all the subjects of the IgAN group and healthy volunteers. The blood samples were either transferred to a purple-capped tube containing 8 mL of EDTA (anticoagulant) for isolation of peripheral blood mononuclear cells (PBMCs) or to a yellow-capped tube containing 2 mL of procoagulant for separation of plasma (1000 g for 10 min at room temperature). PBMCs were isolated by Ficoll–Hypaque density-gradient centrifugation (density, 1.077 g/mL; 800 × *g* for 30 min at room temperature). Freshly isolated PBMCs were washed thrice with Hank’s physiological solution (Beyotime, Jiangsu, China). The percentage cell viability of PBMCs was determined by trypan blue assay. The PBMCs were then split into two parts, one for estimation of total RNA, and the other for estimation of total protein. All specimens were clearly labeled with all the information and placed in an ultra-low-temperature refrigerator at − 80 °C.

### Immunohistochemistry

Following are the steps for immunohistochemistry: Deparaffinization of the paraffin sections of renal tissues followed by hydration, blocking using an endogenous peroxidase, antigen retrieval, incubation in xeno- and serum-free medium, addition of primary antibody (4 °C, overnight), and then incubation with secondary antibody (37 °C, 30 min). Finally, DAB was used as chromogen and hematoxylin was used as counterstain. The primary antibodies used are as follows: anti-BTK (1:100, Cell Signaling, MA, USA), anti-pBTK (1:100, Affinity Biosciences, Cincinnati, USA), anti-CD68 (1:200, Arigo, Taiwan, China), p-NF-κB p65 (1:100, Affinity, Cincinnati, USA), TNF-ɑ (1:500, Arigo, Taiwan, China), IL-1β (1:100, Affinity, Cincinnati, USA), C3b (1:100, Abcam, MA, USA), and C4d (1:100, Abcam, MA, USA). The secondary antibody used is PV-6000 (Zsbio, Beijing, China). Sections of renal tissues incubated in PBS were used as negative controls. The Image-Pro-Plus software version 6.0 was used to analyze the immunohistochemical staining.

### Western blot analyses

PBMCs were resolved in lysis buffer with a protease inhibitor (Beyotime, Shanghai, China) at 4 °C for 30 min. They were then centrifuged at 12,500 rpm and 4 °C for 20 min and the supernatant was collected after centrifugation. Protein concentration was measured using Enhanced BCA Protein Assay Kit (Beyotime, Shanghai, China). Next, the protein was added to loading buffer and the mixture was boiled in boiling water for 10 min. The total protein was electro-transferred to PVDF membranes. The membranes were successively incubated with primary antibody and secondary antibody combined with horseradish peroxidase. The protein bands were observed using a chemiluminescence system. The primary antibodies used are as follows: anti-BTK, anti-pBTK (Cell Signaling, MA, USA), NF-κB p65 (Cell Signaling, MA, USA), phospho-NF-κB p65.

### Real-time reverse transcription-polymerase chain reaction (RT-PCR)

Total RNA was extracted from PBMCs using TRIzol reagent (Invitrogen, Carlsbad, CA). The cDNA was synthesized in 20 µL of reaction mixture using Reverse Transcription System (Vazyme Biotech Co, Nanjing, China) at 50 °C for 15 min and 85 °C for 5 s. The quantity of mRNA was determined using AceQ qPCR SYBR Green Master Mix (Vazyme Biotech Co, Nanjing, China). The primers used were—β-actin, Forward primer: 5′-GCGAGAAGATGACCCAGATC-3′, Reverse primer: 5′-GGATAGCACAGCCTGGATA-3′; TNF-α, Forward primer: 5′-GCCTCTGTGCCTTCTTTTGA-3′, Reverse primer: 5′-GCAACCTTTATTTCTCGCCA-3′; IL-1β, Forward primer: 5′-CGACACATGGGATAACGAGGCTT-3′, Reverse primer: 5′-TCTTTCAACACGCAGGACAGGTA-3′; and MCP-1, Forward primer: 5′-CAAACTGAAGCTCGCACTCTCGCC-3′, Reverse primer: 5′-ATTCTTGGGTTCTGGAGTGAGTGTTCA-3′. Amplification was implemented in 20 µL of reaction volume according to the manufacturer’s instructions. The relative mRNA levels were calculated by the $2^{{ - \Delta \Delta C_{{\text{T}}} }}$ method.

### Enzyme-linked immunosorbent assay (ELISA)

Plasma levels of TNF-ɑ, IL-1β, and MCP-1 were determined by ELISA (RayBiotech, Guangzhou, China) following the manufacturer’s protocol.

### Statistical analyses

The IBM SPSS Statistics software version 23 was used to analyze the statistical data. All the data conforming to normal distribution were expressed as Mean ± SD. The *t*-test was used to compare the difference between two independent samples, namely the control and IgAN groups. *p* < 0.05 indicated that the difference was statistically significant.

## Results

### Baseline clinical and pathological characteristics of study subjects

The clinical characteristics of 63 patients with primary IgAN recruited as IgAN group comprising 23 males and 40 females are shown in Table 1. The pathological parameters of the IgAN group are described in Table 2. In the IgAN group, 29 patients (46.03%) had mesangial hypercellularity (M1), 24 (39.68%) had endocapillary hypercellularity (E1), 39 (61.90%) had segmental glomerulosclerosis (S1), 19 (30.16%) had renal tubular atrophy/interstitial fibrosis (T1/2), and 21 (34.92%) had cellular or fibrocellular crescents (C1/2). According to Katafuchi semi-quantitative scoring method, total score was 8.64 ± 4.14, glomerular score was 4.00 (2.50, 6.00), tubulointerstitial score was 3.00 (2.00, 4.00), and vessel score was 1.00 (1.00, 2.50).

**Table 1:** Clinical characteristics of IgA patients [Mean ± SD, M (P25, P75)]

Clinical indicators	IgAN (*n* = 63)
Age (years)	37.00 (28.00, 45.25)
Gender, male/female	23/40
SBP (mmHg)	126.13 ± 15.59
DBP (mmHg)	81.00 (75.50, 90.50)
MAP (mmHg)	96.67 (87.67, 104.67)
Leukocyte count (/nL)	7.01 (5.94, 7.91)
Monocyte count (/μL)	382.71 ± 150.80
Proteinuria (g/24 h)	0.91 (0.47, 1.54)
Hematuria (/HPF)	20.00 (6.00, 56.60)
Alb (g/L)	40.15 ± 4.58
Scr (μmol/L)	72.70 (55.65,101.28)
BUN (mmol/L)	5.51 ± 1.88
eGFR (ml/min)	99.50 (78.00, 128.00)
UA (mmol/L)	371.62 ± 95.81
Serum IgA (g/L)	2.94 (2.54, 3.76)
Serum C3 (g/L)	1.08 ± 0.17

*Alb* albumin, *BUN* blood urea nitrogen, *DBP* diastolic blood pressure, *eGFR* estimated glomerular filtration rate, *MAP* mean arterial pressure, *SBP* systolic blood pressure, *Scr* serum creatinine, *UA* uric acid

**Table 2:** Histological features of patients with IgA nephropathy

Histological features	
Oxford classification (MEST-C) [Mean ± SD, *n* (%)]	
Number of glomerulus	19.64 ± 9.53
M1	29.00 (46.03%)
E1	24.00 (39.68%)
S1	39.00 (61.90%)
T1/2	19.00 (30.16%)
C1/2	21.00 (34.92%)
Katafuchi semi-quantitative criteria [Mean ± SD, M (P25, P75)]	
Total scores	8.64 ± 4.14
Glomerulus scores	4.00 (2.50,6.00)
Tubulointerstitial scores	3.00 (2.00, 4.00)
Vessel scores	1.00 (1.00, 2.50)

M0: < 50% of glomeruli showing mesangial hypercellularity; M1: > 50% of glomeruli showing; E0: no endocapillary hypercellularity; E1: any glomeruli showing endocapillary hypercellularity; S0: no segmental glomerulosclerosis; S1: present in any glomeruli; T0: 0–25% of cortical area present tubular atrophy/interstitial fibrosis; T1/2: > 26% of cortical area present above feature; C0: no crescents; C1/2: present in any glomeruli

### Expression of CD68 and BTK in renal tissues

**Fig. 1:**
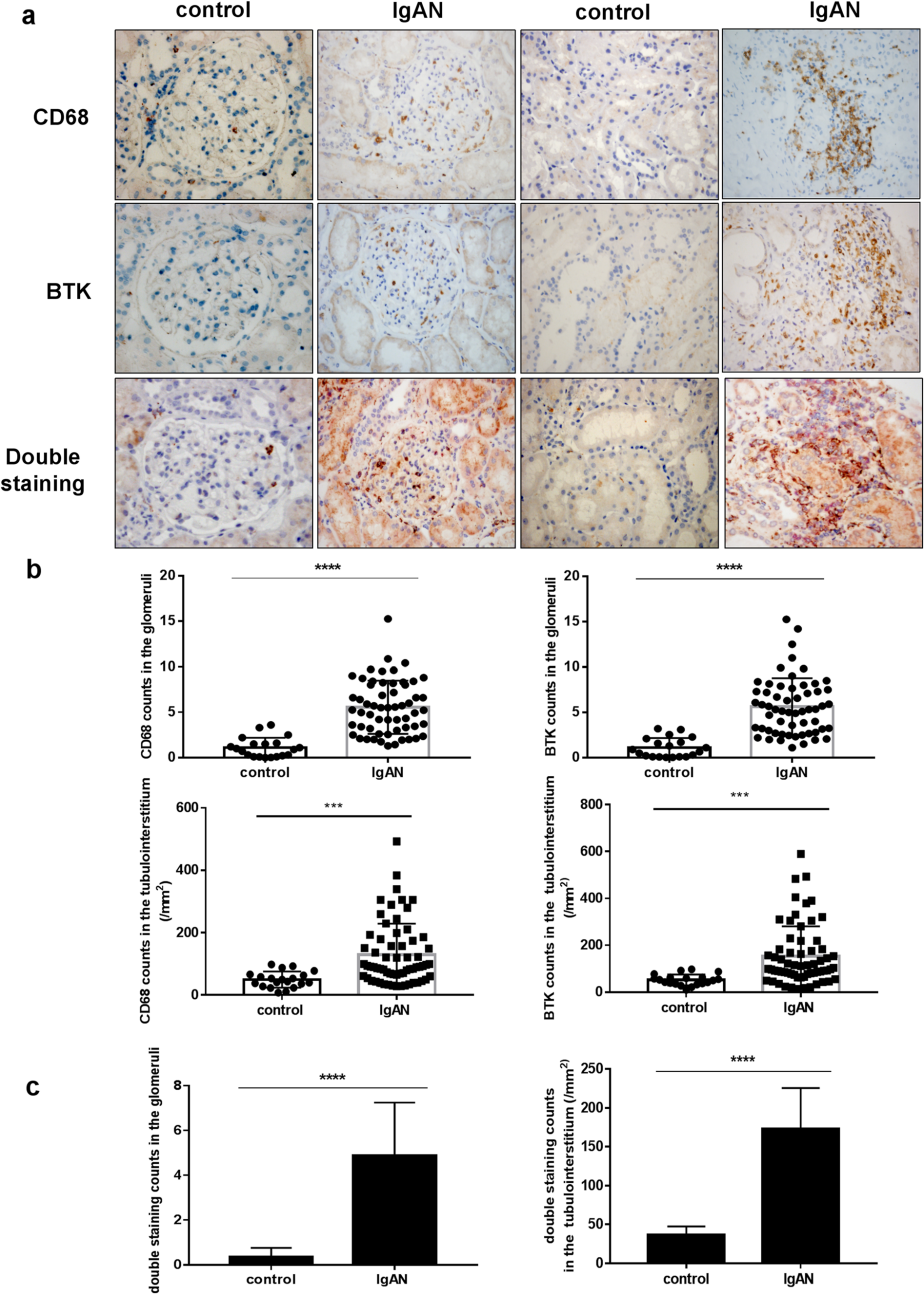
Expression of CD68 and BTK in renal tissues (× 400). **a** The single and double staining of CD68 and BTK in peritumoral normal renal tissues and IgAN renal tissues. **b** The count of single staining CD68 and BTK in glomeruli and tubulointerstitium (****p < 0.0001). **c** The count of double staining for CD68 and BTK in glomeruli and tubulointerstitium (****p < 0.0001)

The expression levels of CD68 and BTK in the paraffin sections of human renal tissues were measured by immunohistochemical staining using anti-CD68 and anti-BTK antibodies. There was no obvious positive staining for CD68 and BTK in the glomeruli and tubulointerstitia of the peritumoral and normal renal tissues. However, the infiltration of CD68 and BTK was significantly increased in the IgAN group. To ascertain whether CD68 expression is associated with BTK expression in renal tissues, we performed double staining for CD68 and BTK in paired tissues sections. The paired sections showed that the expression levels of CD68 and BTK were consistent in their location and number in the glomeruli and tubulointerstitium of the renal tissues of patients with IgAN (****p < 0.0001, Fig. 1a–c).

### Relationship between expression of BTK in renal tissues and Oxford Classification in patients with IgAN

**Fig. 2:**
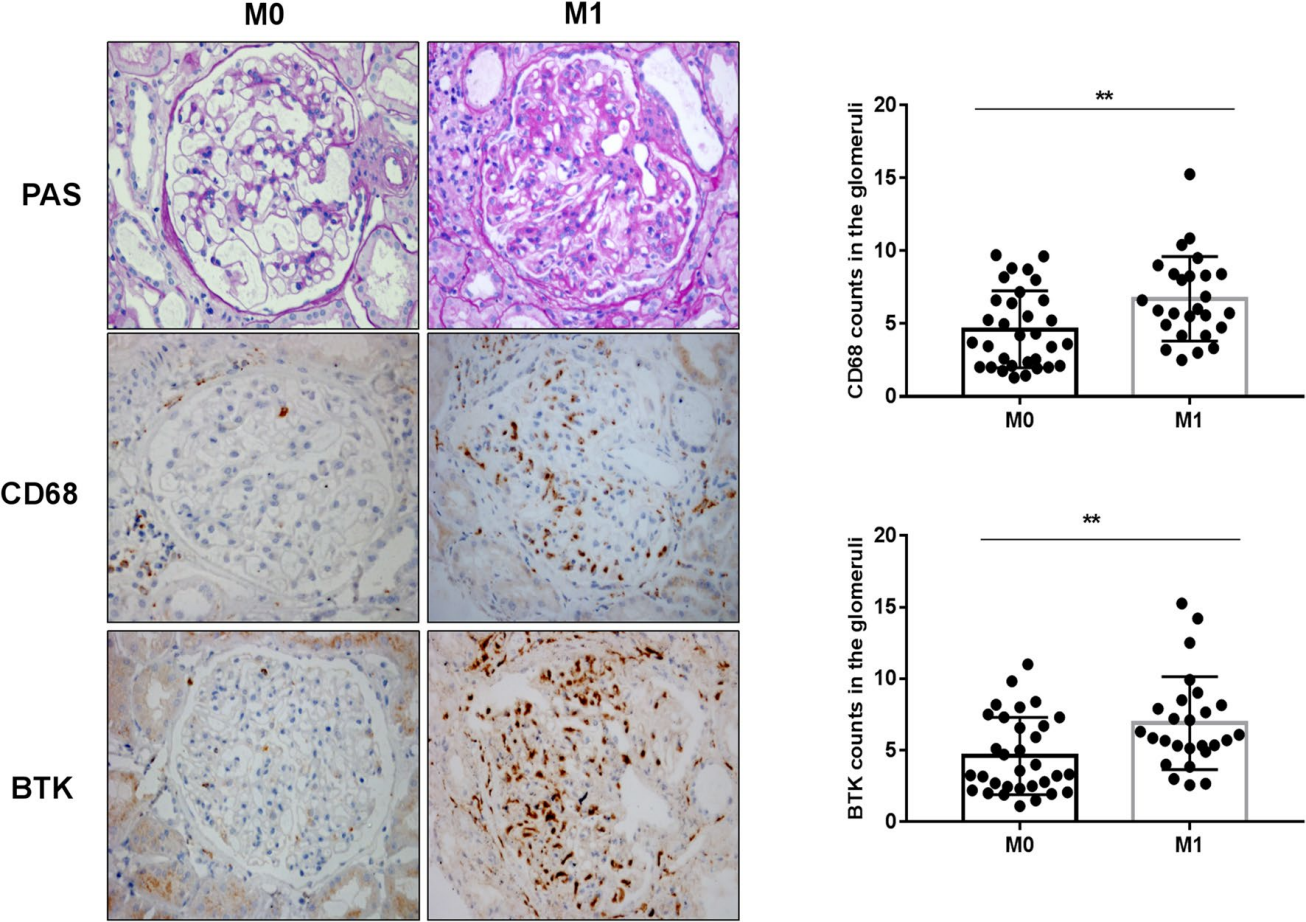
Relationship between expression of BTK in renal tissues and Oxford Classification (M) in patients with IgAN (× 400): staining of the sequential sections of renal tissues for PAS, CD68, and BTK in M0 and M1 of Oxford Classification and the count of CD68 and BTK in M0 and M1 of the classification (**p < 0.05)

**Fig. 3:**
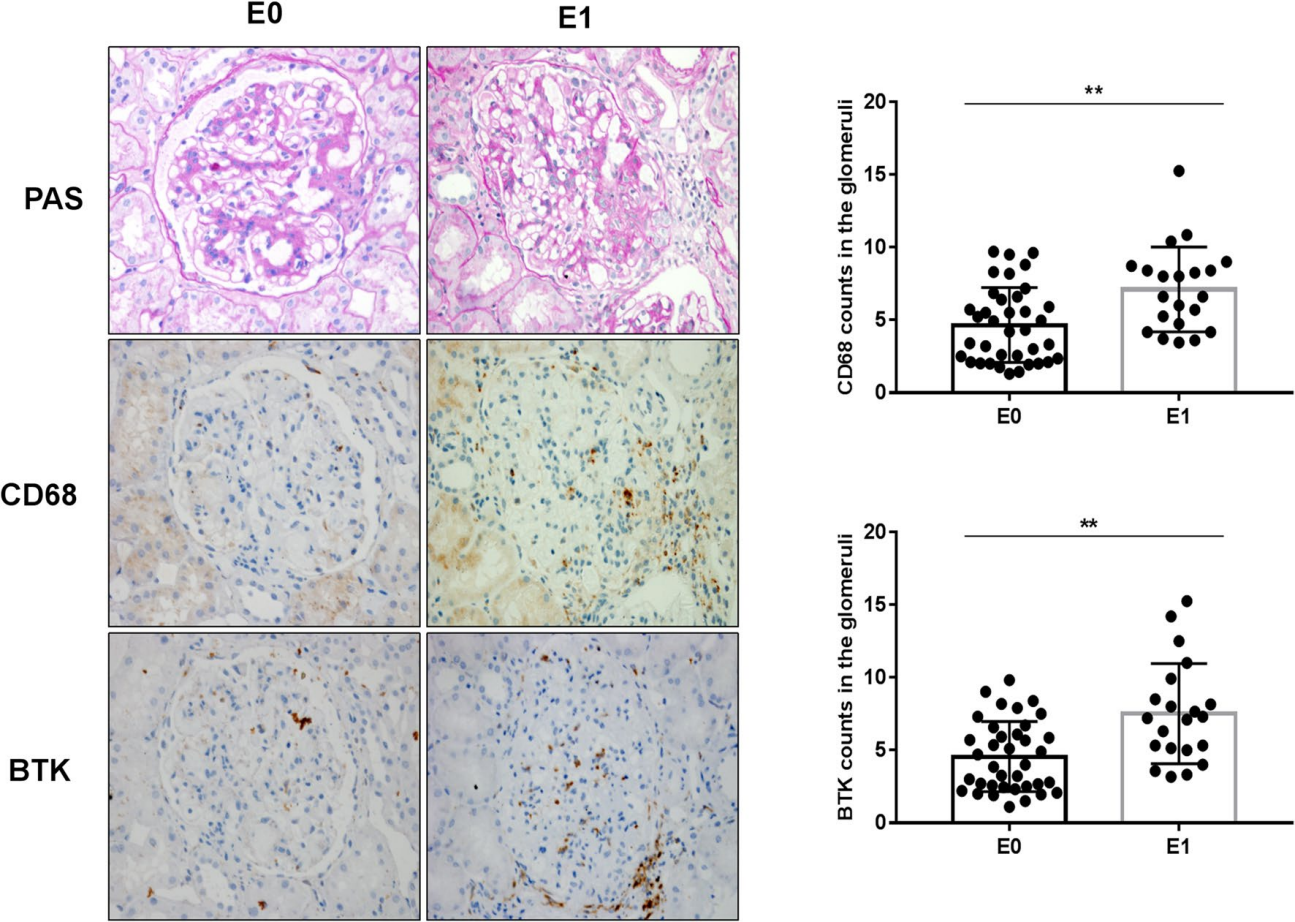
Relationship between expression of BTK in renal tissues and Oxford Classification (E) in patients with IgAN (× 400): staining of the sequential sections of renal tissues for PAS, CD68, and BTK in E0 and E1 of Oxford Classification and the count of CD68 and BTK in E0 and E1 of the classification (**p < 0.05)

**Fig. 4:**
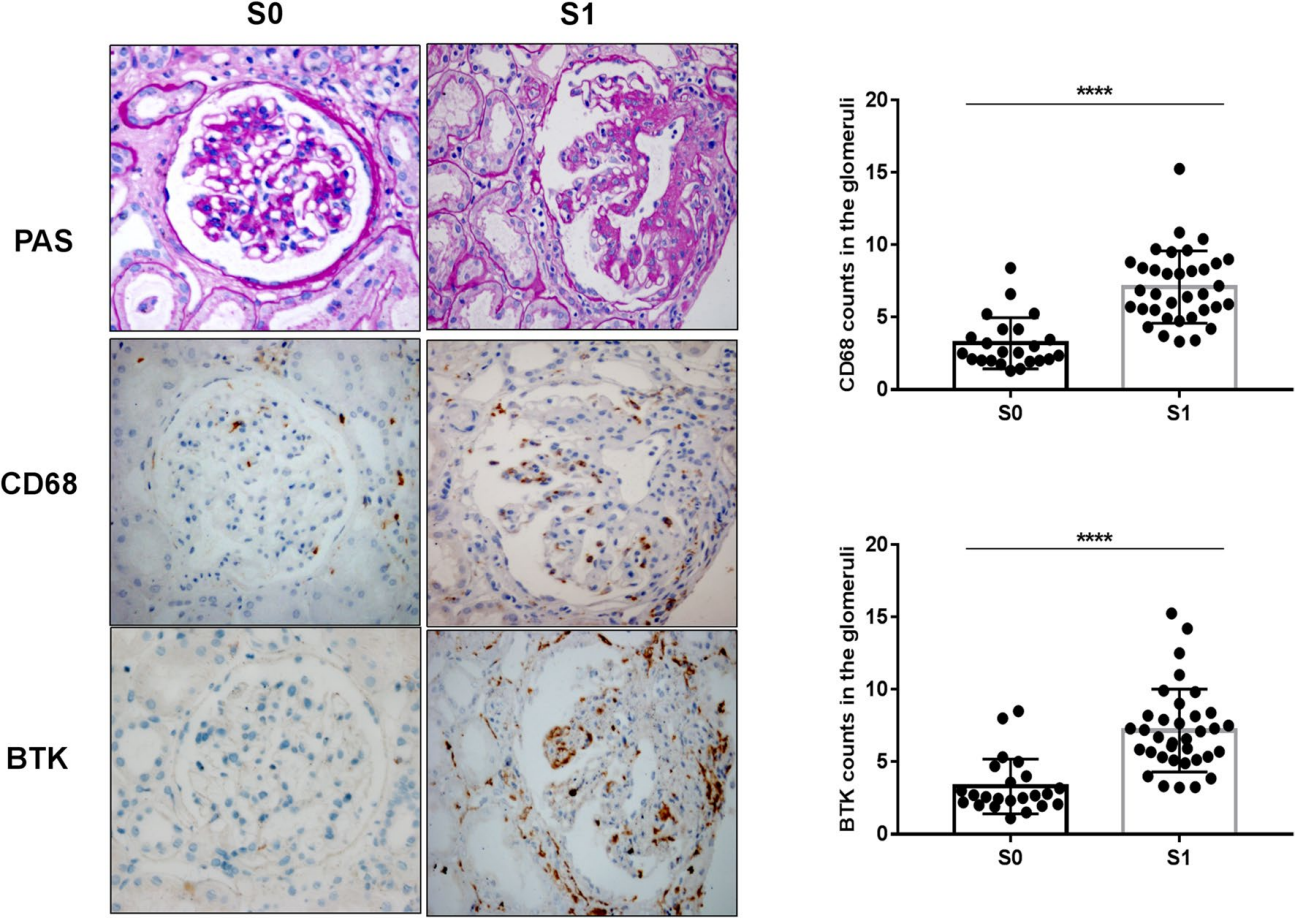
Relationship between expression of BTK in renal tissues and Oxford Classification (S) in patients with IgAN (× 400): staining of the sequential sections of renal tissues for PAS, CD68, and BTK in S0 and S1 of Oxford Classification and the count of CD68 and BTK in S0 and S1 of the classification (****p < 0.0001)

**Fig. 5:**
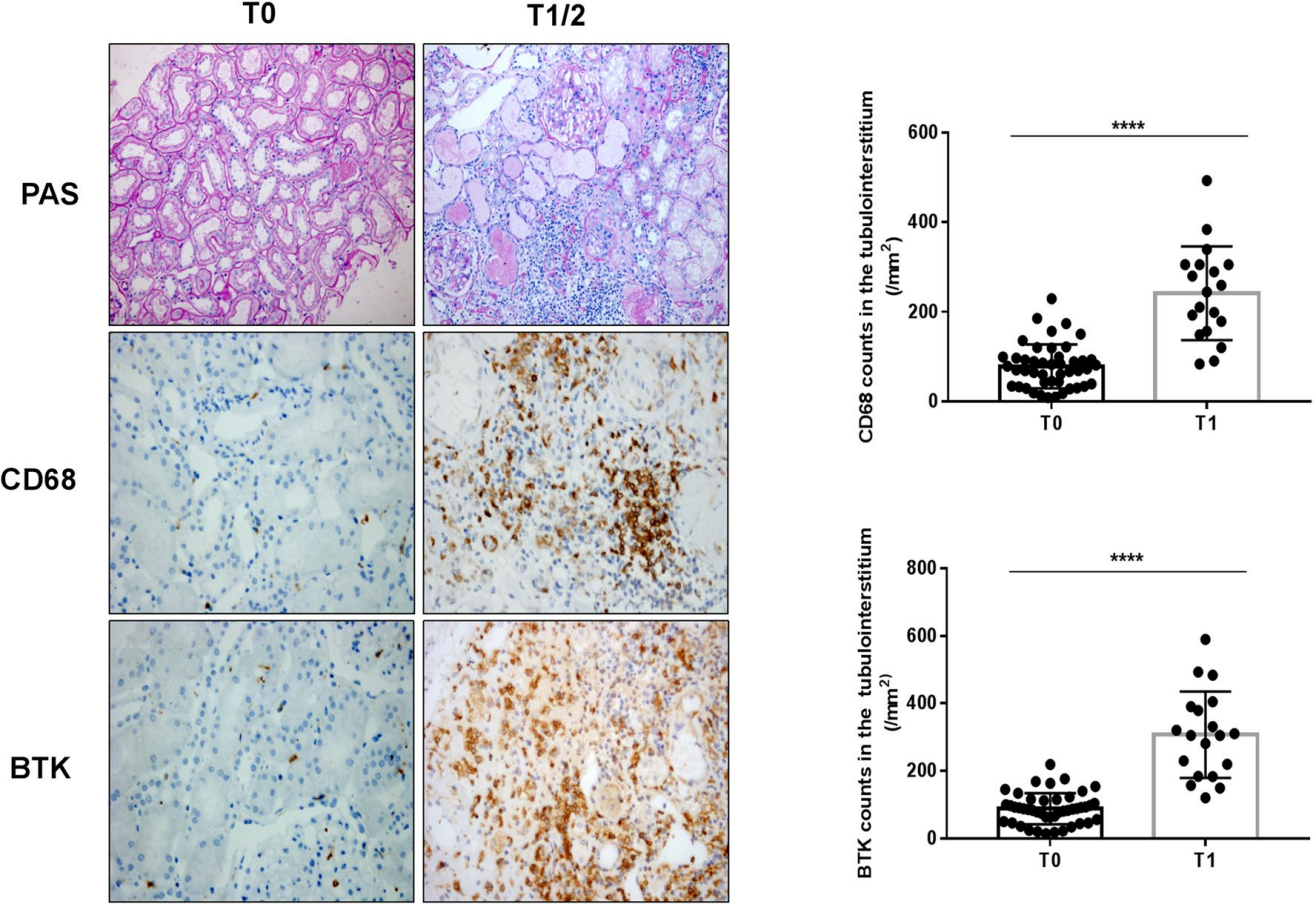
Relationship between expression of BTK in renal tissues and Oxford Classification (T) in patients with IgAN (× 400): staining of the sequential sections of renal tissues for PAS, CD68, and BTK in T0 and T1-2 of Oxford Classification and the count of CD68 and BTK in T0 and T1-2 of the classification (****p < 0.0001)

**Fig. 6:**
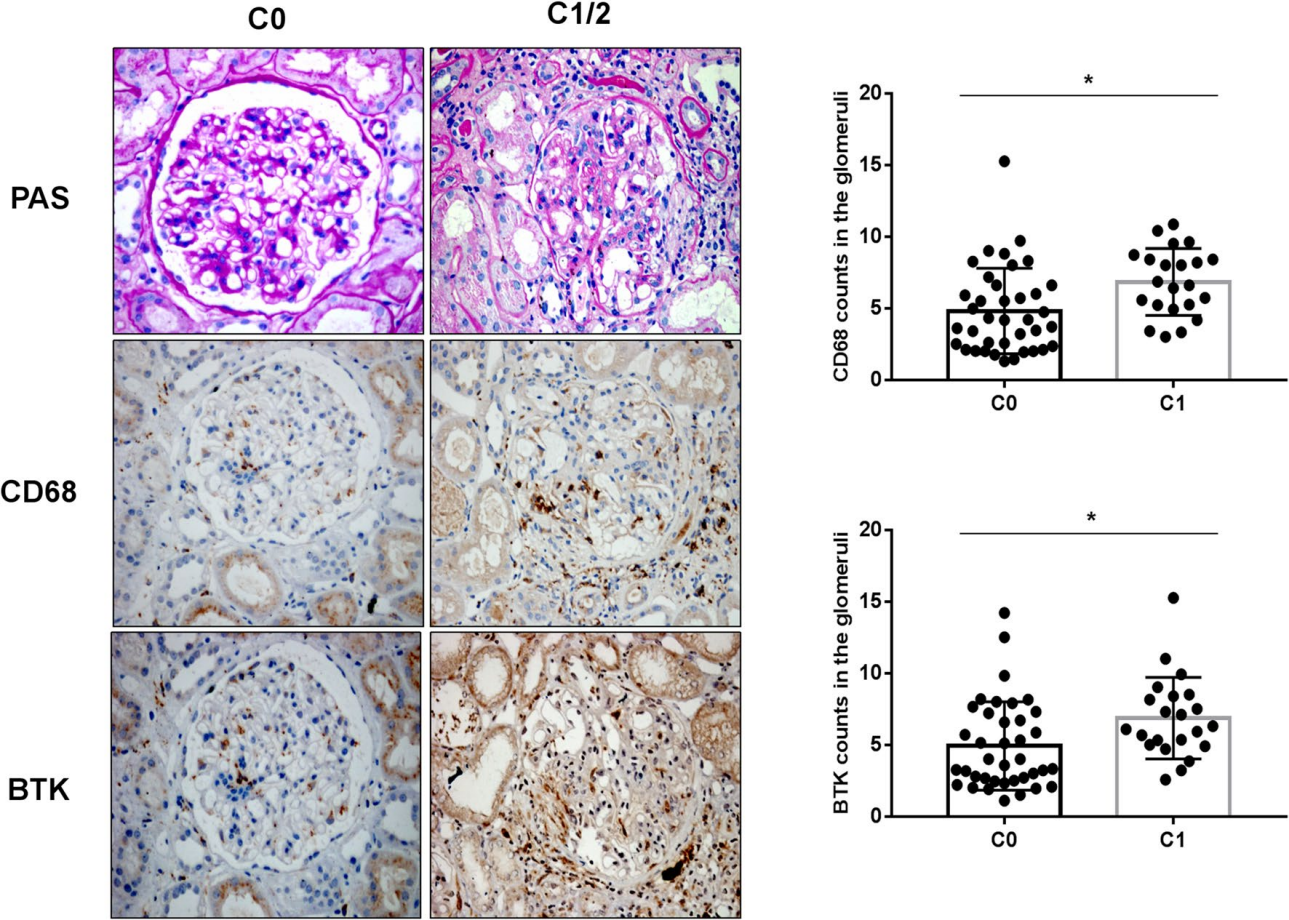
Relationship between expression of BTK in renal tissues and Oxford Classification (C) in patients with IgAN (× 400): staining of the sequential sections of renal tissues for PAS, CD68, and BTK in C0 and C1–2 of Oxford Classification and the count of CD68 and BTK in C0 and C1–2 of the classification (*p < 0.05)

To investigate the correlation between CD68, BTK expression in IgAN renal tissues and Oxford Classification (MEST-C), we performed sequential staining of IgAN renal tissue section for Periodic Acid-Schiff (PAS), CD68, and BTK. The results indicated there were statistically significant differences between M0 and M1 (**p < 0.05; Fig. 2a, b), E0 and E1 (**p < 0.05; Fig. 3a, b), S0 and S1 (****p < 0.0001; Fig. 4a, b), T0 and T1–2 (****p < 0.0001; Fig. 5a, b), and C0 and C1-2 (*p < 0.05; Fig. 6a, b) in Oxford Classification (MEST-C).

### Expression of CD68, BTK in renal tissues and Katafuchi semi-quantitative score of patients with IgAN

**Fig. 7:**
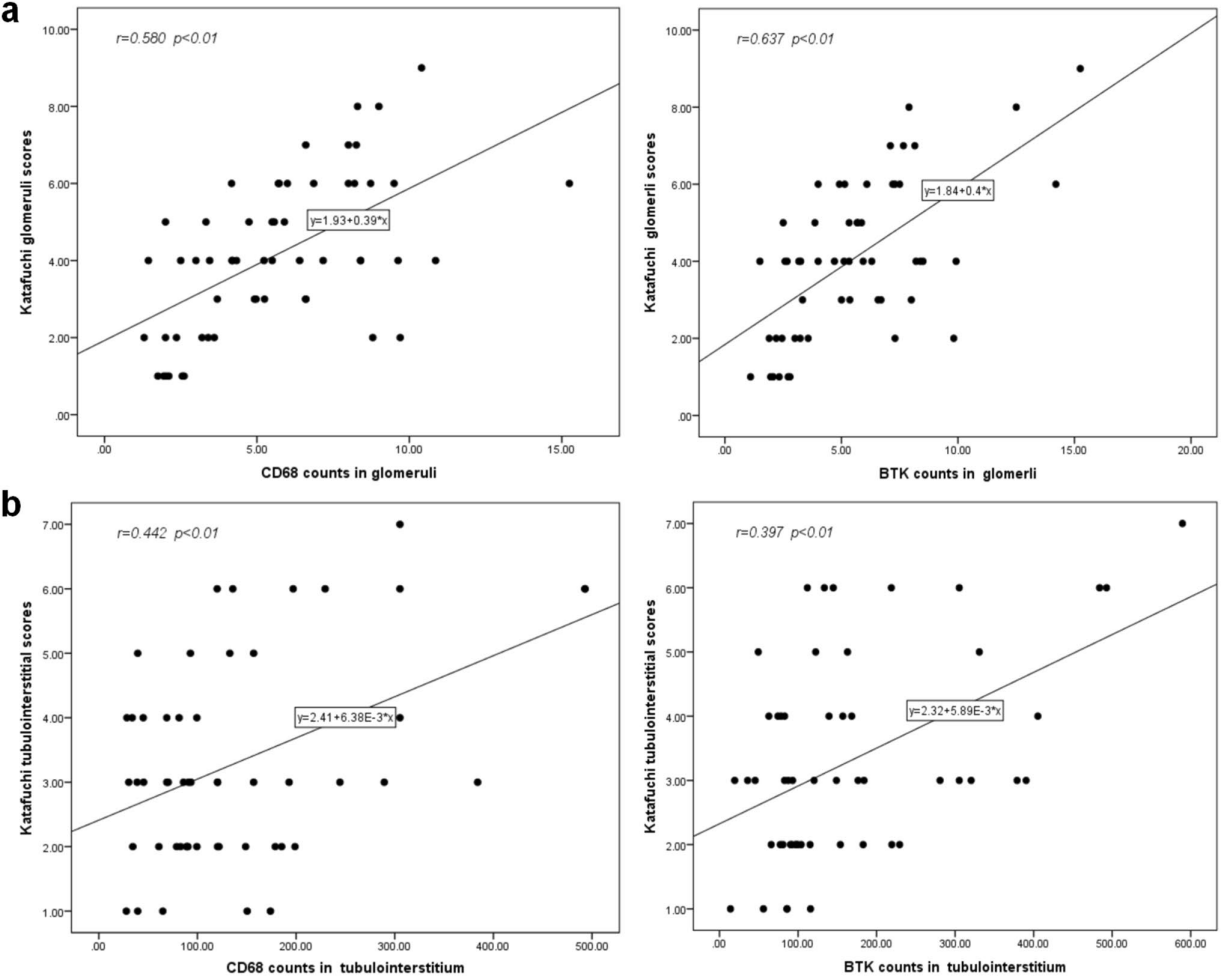
Correlation analysis between CD68, BTK expression in renal tissue and Katafuchi semi-quantitative score. **a** The expression of CD68 and BTK in glomeruli were positively correlated with Katafuchi semi-quantitative glomerular scores (*r* = 0.580 and 0.637, **p < 0.05). **b** The expression of CD68 and BTK in renal tubulointerstitium were positively correlated with Katafuchi semi-quantitative tubulointerstitial scores (*r* = 0.442 and 0.489, **p < 0.05)

As shown in Fig. 7a, b, the expression of CD68 and BTK in glomeruli was positively correlated with Katafuchi semi-quantitative glomerular scores (*r* = 0.580 and 0.637, **p < 0.05). And the expression of CD68 and BTK in renal tubulointerstitium was positively correlated with Katafuchi semi-quantitative tubulointerstitial scores (*r* = 0.442 and 0.489, **p < 0.05).

### Expression of pBTK and p-NF-κB p65 in renal tissues

**Fig. 8:**
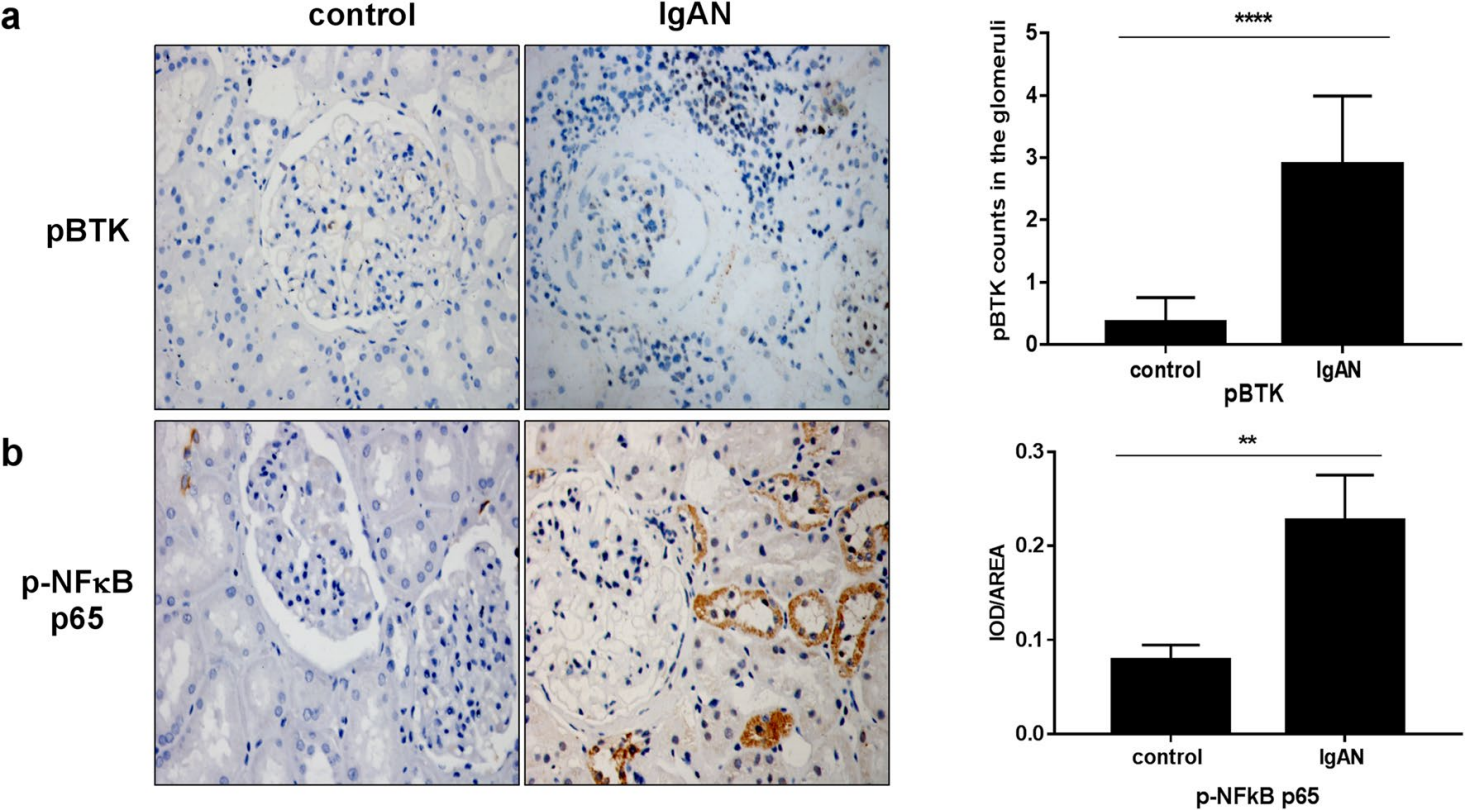
Expression of pBTK and p-NF-κB p65 in renal tissues (× 400). **a** The staining of p-BTK and p-NF-κB p65 in peritumoral normal renal tissues and IgAN renal tissues. **b** The count of pBTK and p-NF-κB p65 in renal kidney (*p < 0.05)

There was significant staining for p-BTK and p-NF-κB p65 in IgAN renal tissues (Fig. 8a, b,*p < 0.05). However, there was no distinct staining for p-BTK and p-NF-κB p65 in the peritumoral normal renal tissues. These results suggest that p-BTK and p-NF-κB p65 might not be activated in normal tissues.

### Expression of BTK in renal tissues and complement levels of C3b and C4d in patients with IgAN

**Fig. 9:**
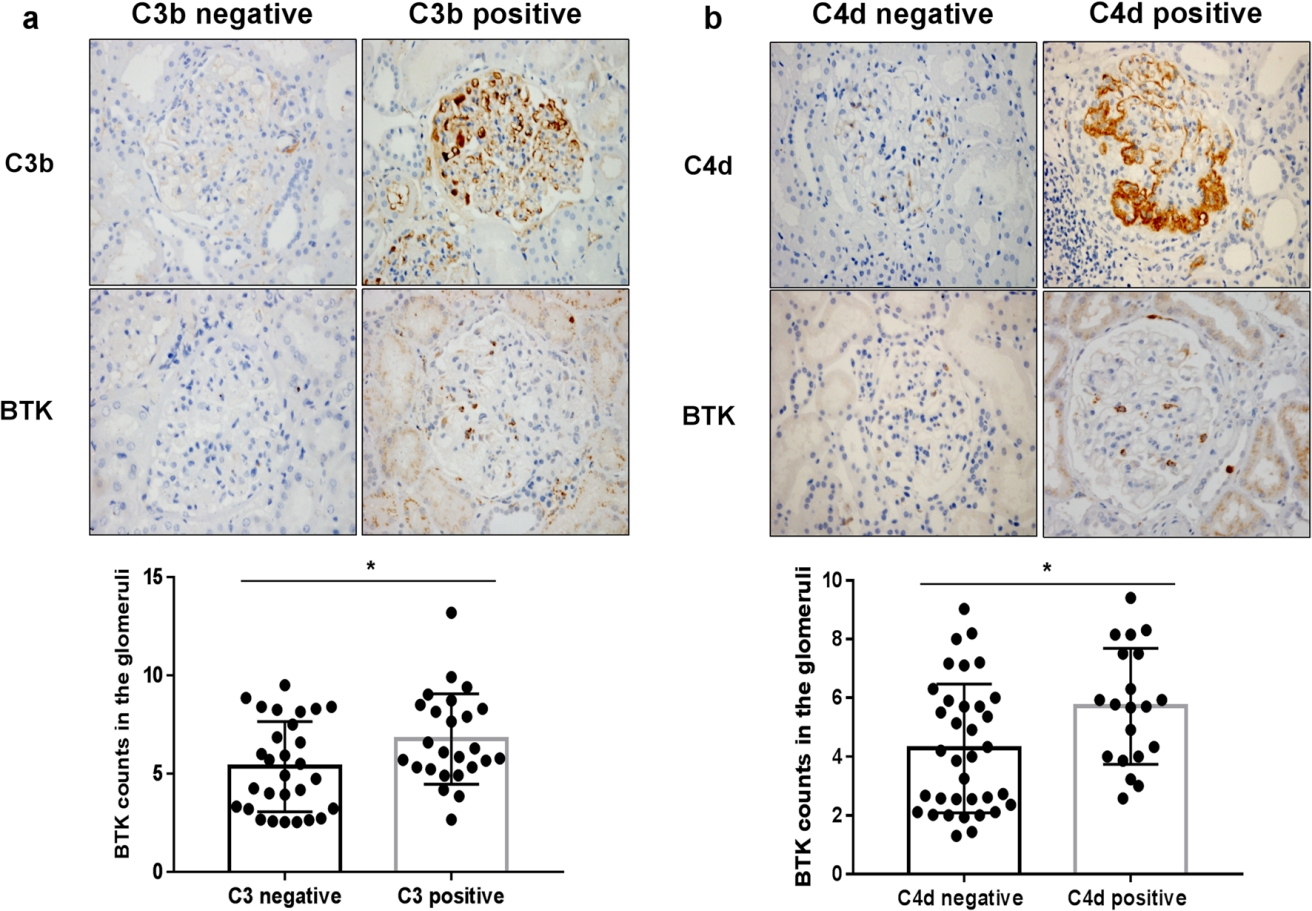
Relationship between expression of BTK in renal tissues and complement levels of C3b and C4d in renal tissues of patients with IgAN (× 400). **a** The expression of BTK was compared between C3b-positive and C3b-negative IgAN renal tissues (*p < 0.05). **b** The expression of BTK was compared between C4d-positive and C4d-negative IgAN renal tissues (*p < 0.05)

As shown in Fig. 9a, b, we investigated the correlation between BTK expression and complement levels of C3b and C4d in IgAN renal tissues. The results revealed that the expression of BTK was significantly higher in C3b-positive and C4d-positive IgAN renal tissues (*p < 0.05).

### Expression of TNF-ɑ and IL-1β in renal tissues

**Fig. 10:**
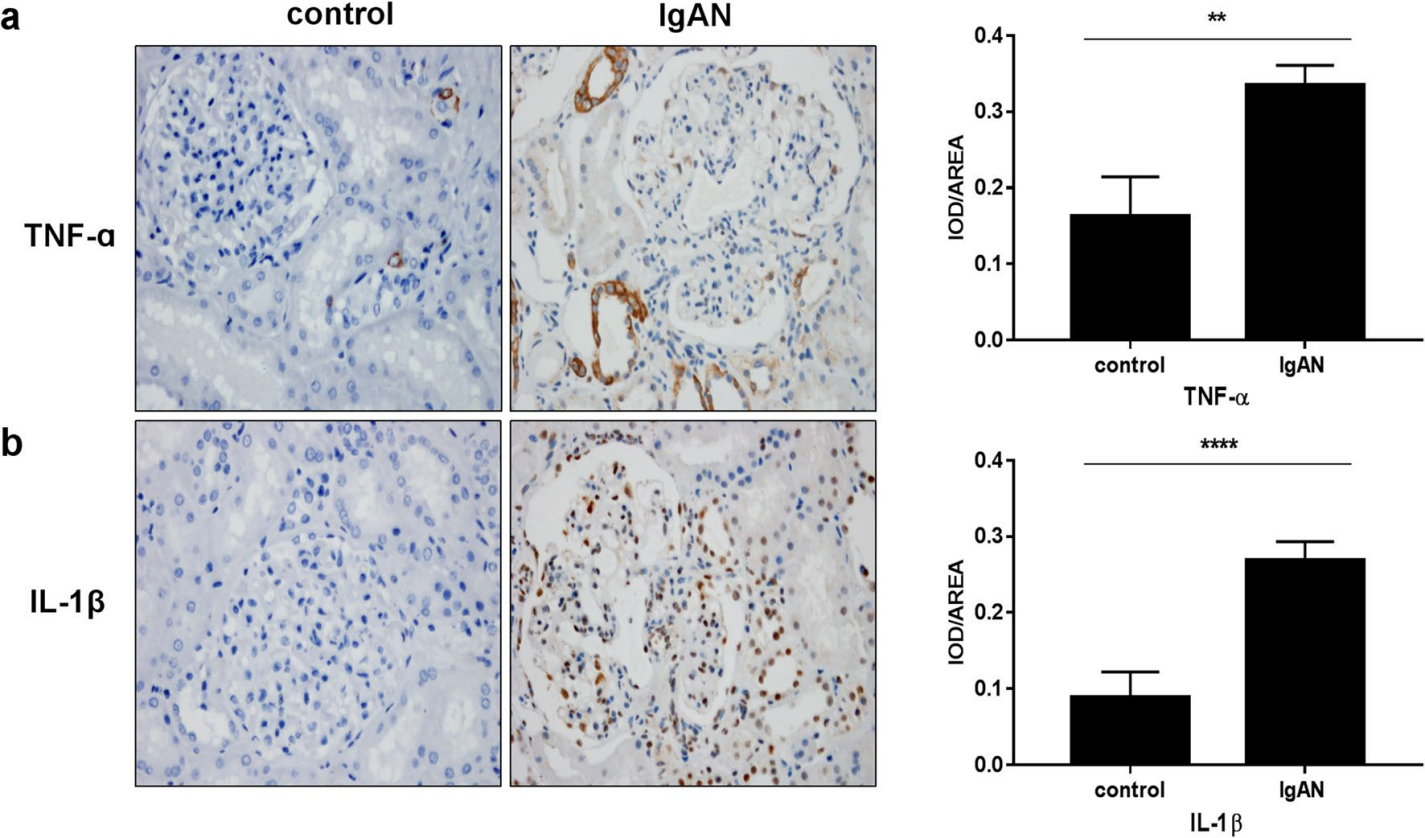
Expression of TNF-ɑ and IL-1βin in renal tissues (× 400). **a** The staining of TNF-ɑ in peritumoral normal renal tissues and IgAN renal tissues (**p < 0.05). **b** The staining of IL-1β in peritumoral normal renal tissues and IgAN renal tissues (****p < 0.0001)

There was a significant increase in the levels of proinflammatory cytokines in renal tissues of patients with IgAN patients compared to that in the control group. As shown in Fig. 10a, b, the expression of TNF-ɑ, IL-1β in IgAN renal tissues was evidently accelerated compared to that in peritumoral normal renal tissues (**p < 0.05).

### Expression of BTK in renal tissues and clinical characteristics of patients with IgAN

**Fig. 11:**
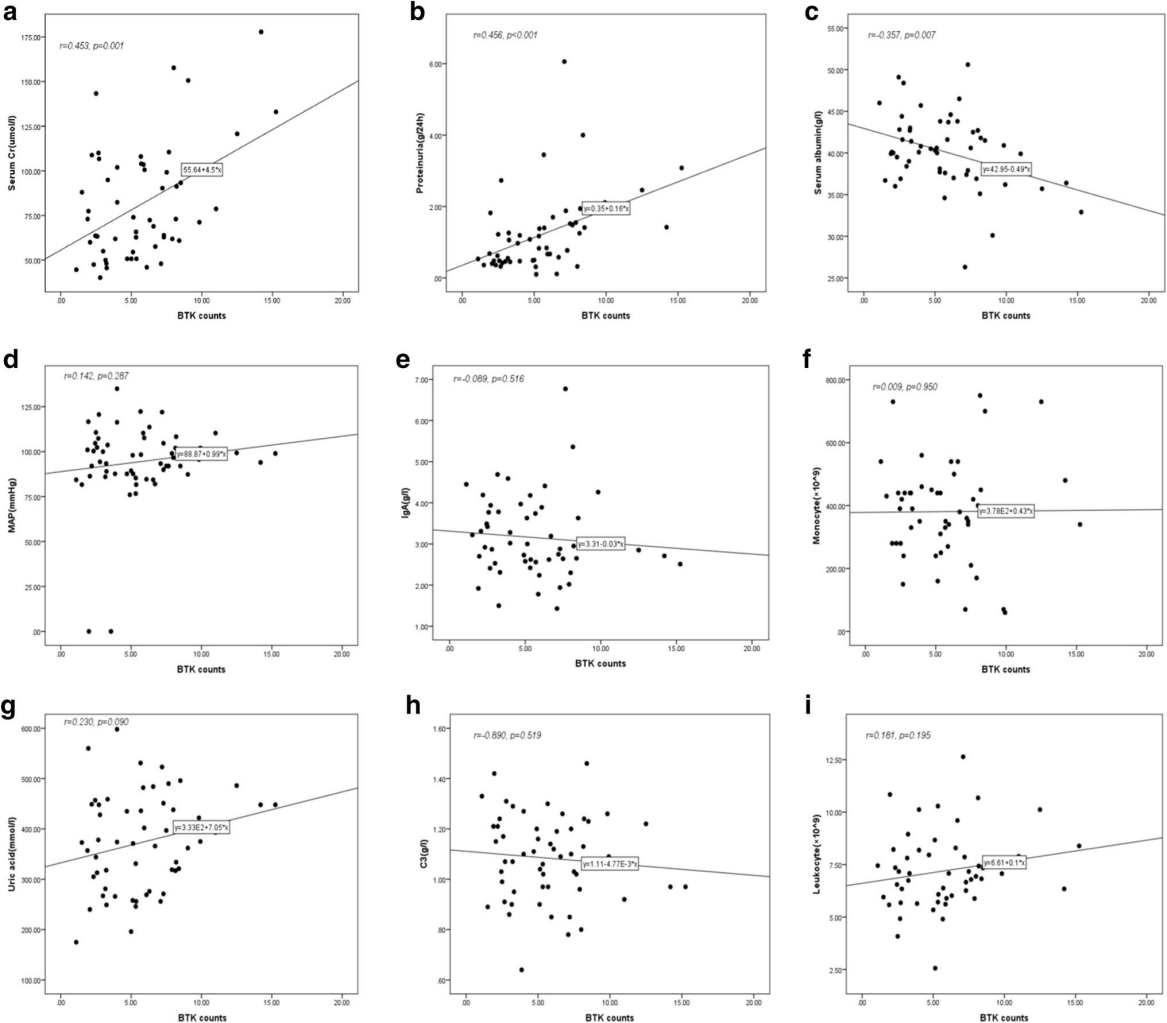
Expression of BTK in renal tissues and clinical characteristics of patients with IgAN. **a** The expression of BTK was positively correlated with Scr (*r* = 0.453; ***p < 0.001). **b** The expression of BTK was positively correlated with the levels of 24-h urine protein (*r* = 0.375; ***p < 0.001). **c** The expression of BTK was negatively correlated with Alb (*r* = 0.456; ***p < 0.001). **d**–**i** The correlation between BTK expression and MAP, leukocytes, monocytes, UA, serum IgA, and serum C3 (*r* = 0.142, 0.181, 0.009, 0.230, 0.089, 0.890, respectively; *p* > 0.05)

As shown in Fig. 11a–c, the expression of BTK was positively correlated with 24-h urine protein and serum creatinine levels (Scr) (*r* = 0.456 and 0.453 respectively, ***p < 0.001) and negatively correlated with serum albumin(Alb) (*r* = 0.357, **p < 0.05). However, as shown in Fig. 11d–i, there was no correlation between BTK expression and mean arterial pressure (MAP), number of leukocytes and monocytes, and levels of uric acid (UA), serum IgA, and serum C3 ( *p* > 0.05).

### Expression of pBTK and p-NF-κB p65 in PBMCs

**Fig. 12:**
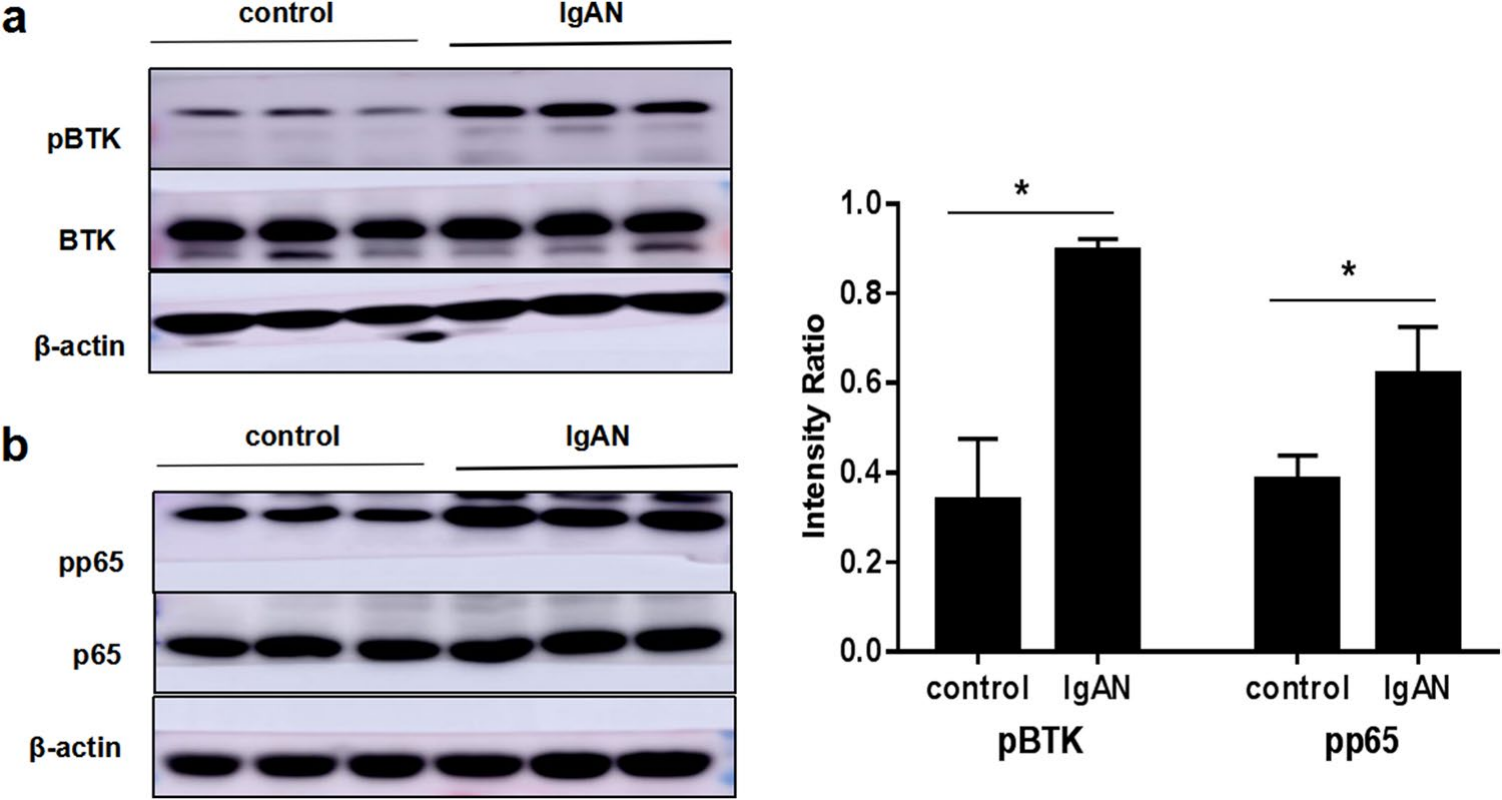
Expression of pBTK and p-NF-κB p65 in PBMCs. **a** The level of pBTK was detected by western blotting in PBMCs of healthy volunteers and patients with IgAN (**p < 0.05). **b** The level of NF-κB p65 was detected by western blotting in PBMCs of healthy volunteers and patients with IgAN (*p < 0.05)

To further explore the effect of BTK on the inflammatory mechanism of IgAN, we evaluated the expression of p-BTK and p-NF-κB p65 by western blotting. The results demonstrated that the levels of p-BTK and p-NF-κB p65 proteins were evidently higher in PBMCs of patients with IgAN compared to that in normal controls (*p < 0.05, Fig. 12a, b).

### Expression of TNF-ɑ, IL-1β, and MCP-1 in PBMCs and plasma

**Fig. 13:**
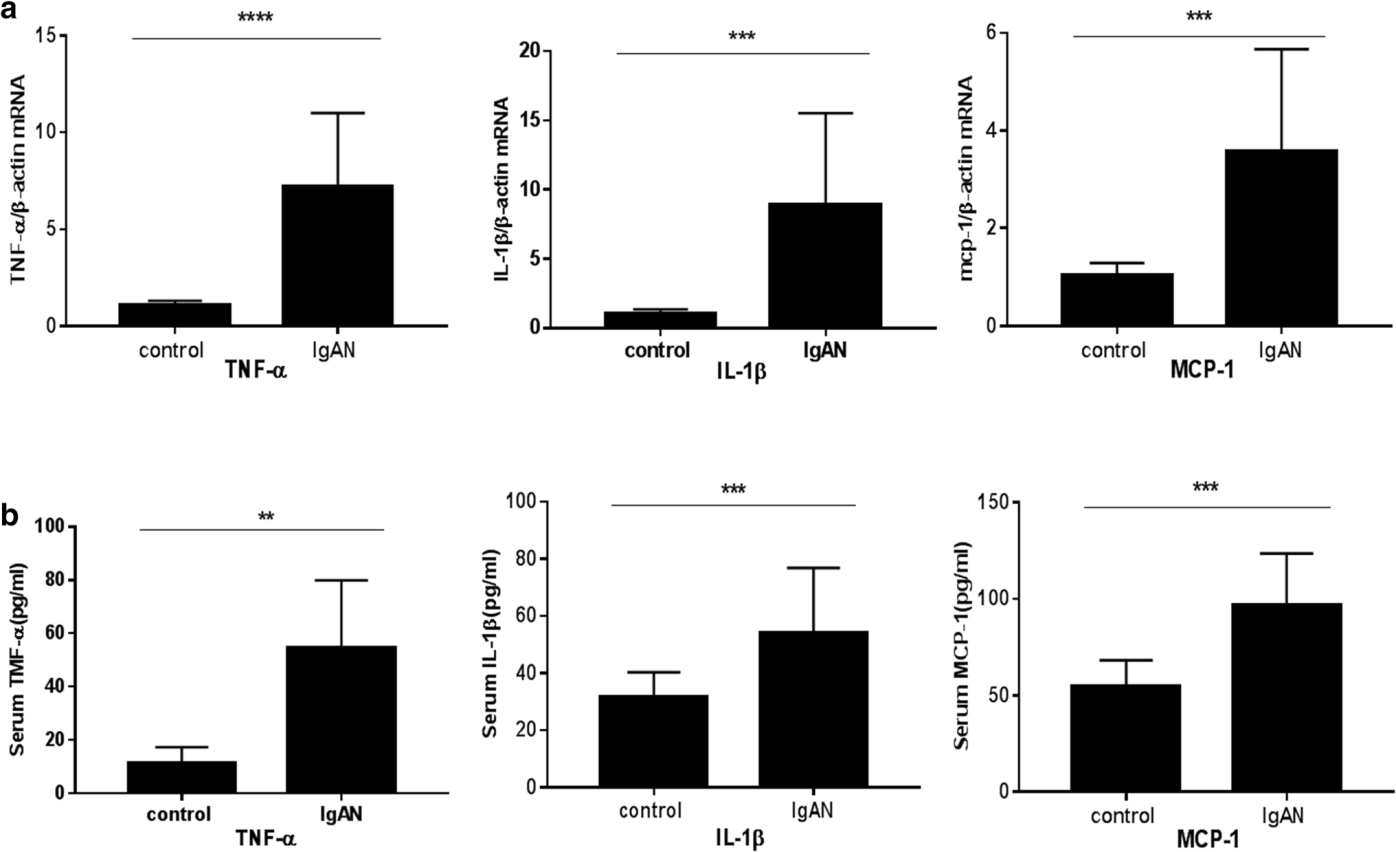
Expression of TNF-ɑ, IL-1β and MCP-1 in PBMCs and plasma. **a** The levels of TNF-ɑ, IL-1β, and MCP-1 in PBMCs were detected by RT-PCR (***p < 0.001). **b** The levels of TNF-ɑ, IL-1β, and MCP-1 in plasma were detected by ELISA (*p < 0.05)

There was a significant increase in the levels of both proinflammatory cytokines in PBMCs and plasma of patients with IgAN patients compared to that in the control group. As shown in Fig. 13a, the mRNA levels of TNF-ɑ, IL-1β, and MCP-1 in were higher in the IgAN group than those in control volunteers (***p < 0.001). Moreover, the results of ELISA indicated that levels of TNF-ɑ, IL-1β, and MCP-1 were markedly increased in the IgAN group (**p < 0.05, Fig. 13b).

## Discussion

In this study, renal biopsy tissues and blood samples from 63 patients were used to systematically analyze the expression of macrophage BTK in IgA nephropathy. The results showed that the expression of CD68 and BTK was markedly increased in patients with IgAN, and while examining the renal biopsies, we found that there is a correlation between BTK expression and Updated Oxford Classification, complement and serum creatinine levels, and proteinuria and albumin. Additionally, the expression of BTK in IgAN patients with mesangial hypercellularity, endocapillary hypercellularity, segmental glomerulosclerosis, tubular atrophy/interstitial fibrosis, and crescents was significantly higher than IgAN patients without those histological manifestations.

In recent years, more and more studies have been conducted on the Splenic tyrosine kinase–Bruton’s tyrosine kinase axis mediating immune-related glomerulonephritis. IgA immune complexes captured by FcɑRI induce SYK and BTK activation, which causes proliferation of glomerular mesangial cells and release of proinflammatory and fibrosis factors, finally leads to the progression of IgAN [21]. It has been reported in the literature that immunohistochemical staining was used to stain the renal tissue of patients with IgA nephropathy, and the results showed that the expression of total SYK and phosphorylated SYK was significantly increased in the glomerulus [22]. Moreover, activated STK is involved in the downstream signaling of IgAN and can be used as a therapeutic target [23]. Therefore, we have discussed the mechanism of BTK in the progression of IgAN in this study.

This study found that the expression of BTK in macrophages was related to that of pathological indicators, the Oxford Classification and Katafuchi semi-quantitative score, and BTK expression was more obvious in patients with severe pathological degree. The expressions of pBTK and PP65 in the pathological tissues were significantly higher than those in normal renal tissues, suggesting that macrophages BTK may mediate the occurrence and progression of renal inflammation by activating the NF-κB signaling pathway. This is consistent with the previous reports that BTK deficiency has been associated with a decrease in the number of monocytes/macrophages in mice [24] and BTK inhibitor RN486 can inhibit macrophage activation and reduce the secretion of cytokines, such as IL-6, IL-8, MMP-7 and MMP-10 [25].

The importance of B-cell activation and immune-complex-mediated Fc receptor activation in the pathogenesis of immune-mediated glomerulonephritis has long been recognized. It has also been reported that BTK + cells in peripheral blood of patients with SLE are associated with disease activity, formation of anti-dsDNA antibodies, proteinuria, and increased C3 levels [26]. Treatment of lupus nephritis using Btk inhibitors showed it prevents the development of proteinuria and reduces the levels of inflammatory cytokines and glomerulosclerosis [27]. In high-glucose condition, BTK inhibitors can downregulate MAPK and NF-κB signaling pathways and decrease the secretion of TNF-α, IL-1β, and MCP-1 [28]. BTK may be an important target in the pathogenesis of IgA nephropathy and is expected to be applied in clinical practice.

Our study also found that the expression of BTK was more obvious in the kidney tissues with positive expression of C3b and C4d, but the mechanism has not been thoroughly discussed. In a study on complement factor H(CFH) and B cell [29], mice lacking CFH leads to altered spleen B-cell development, B cells from CFH-/- mice showed enhanced B-cell receptor(BCR) signaling by increasing phosphorylated Bruton tyrosine kinase (pBTK) and phosphorylated spleen tyrosine kinase (pSYK) levels.CFH could act as a negative regulator of BCR signal and limit autoimmunity. It was reported that the production of immunoglobulin and the increase of complement activation are the key factors in the pathogenesis of Hidradenitis suppurativa (HS). Immunohistochemical staining indicated that C1q, C3b and C4d were significantly expressed in the skin of HS patients [30]. The analysis of signal transduction network shows that the activation of the Bruton’s tyrosine kinase (BTK) and spleen tyrosine kinase (SYK) pathways are potential therapeutic targets for HS.

This study demonstrated that macrophage BTK is involved in the development and progression of IgA nephropathy, but it still has certain limitations. This study failed to identify the direct target, specific mechanism and upstream regulatory factors of BTK in immune inflammation, which need further study.

## Conclusion

In conclusion, these results suggest that macrophage BTK is activated in IgA nephropathy and it is involved in inflammatory response through NF-κB signaling pathway, which implicates that it may be an appropriate therapeutic target, and BTK inhibitors may be a novel treatment strategy for IgA nephropathy.

## Data Availability

All data generated or analyzed during this study are included in this published article.
